# Supplementary material for: A flanking-nicks prime editor (FLICK-PE) system to boost prime editing in dicots
Source: Nat Commun. 2025 Dec 4;17:337. doi: 10.1038/s41467-025-67046-3 (PMC12789627; doi:10.1038/s41467-025-67046-3)
Supplement: Supplementary file 1 — Supplementary Information [file 41467_2025_67046_MOESM1_ESM.pdf]

# Supplementary Information

A flanking-nicks prime editor (FLICK-PE) system to boost prime editing in dicots

**Supplementary Fig. 1 |** Vector structure for PE in soybean.

**Supplementary Fig. 2 |** Design of target sites for testing PE strategies in soybean.

**Supplementary Fig. 3 |** qRT–PCR of PE components expressed in transgenic soybean hairy roots.

**Supplementary Fig. 4 |** Editing efficiency mediated by PE3 and PE–ES$^{nick}$ in soybean hairy roots.

**Supplementary Fig. 5 |** Representative chromatograms of PE3 mediated intended editing in soybean hairy roots.

**Supplementary Fig. 6 |** Editing efficiency mediated by dual-nicking strategies at Targets 3 and 5 in soybean hairy roots.

**Supplementary Fig. 7 |** Design of new target sites for testing FLICK-PE in soybean.

**Supplementary Fig. 8 |** Evaluation of off-target effects from FLICK-PE in soybean hairy roots induced by pegRNA 1.

**Supplementary Fig. 9 |** Evaluation of off-target effects from FLICK-PE in soybean hairy roots induced by pegRNA 3.

**Supplementary Fig. 10 |** Evaluation of off-target effects from FLICK-PE in soybean hairy roots induced by pegRNA 5.

**Supplementary Fig. 11 |** Evaluation of off-target effects from FLICK-PE in soybean hairy roots induced by pegRNA 8.

**Supplementary Fig. 12 |** Evaluation of off-target effects from FLICK-PE in soybean hairy roots induced by pegRNA 9.

**Supplementary Fig. 13 |** Evaluation of off-target effects from FLICK-PE in soybean hairy roots induced by pegRNA 10.

**Supplementary Fig. 14 |** Evaluation of off-target effects from FLICK-PE in soybean hairy roots induced by pegRNA 11.

**Supplementary Fig. 15 |** FLICK-PE strategy further relieves the inhibitory effect of the MMR pathway on PE efficiency.

**Supplementary Fig. 16** | Evaluation of off-target effects from FLICK-PE in soybean stable transformation.

**Supplementary Fig. 17 |** Amino-acid sequence alignment of soybean EPSPS homologs EPSPS1a and EPSPS1b.

**Supplementary Fig. 18 |** Design of target sites for testing PE strategies in tobacco.

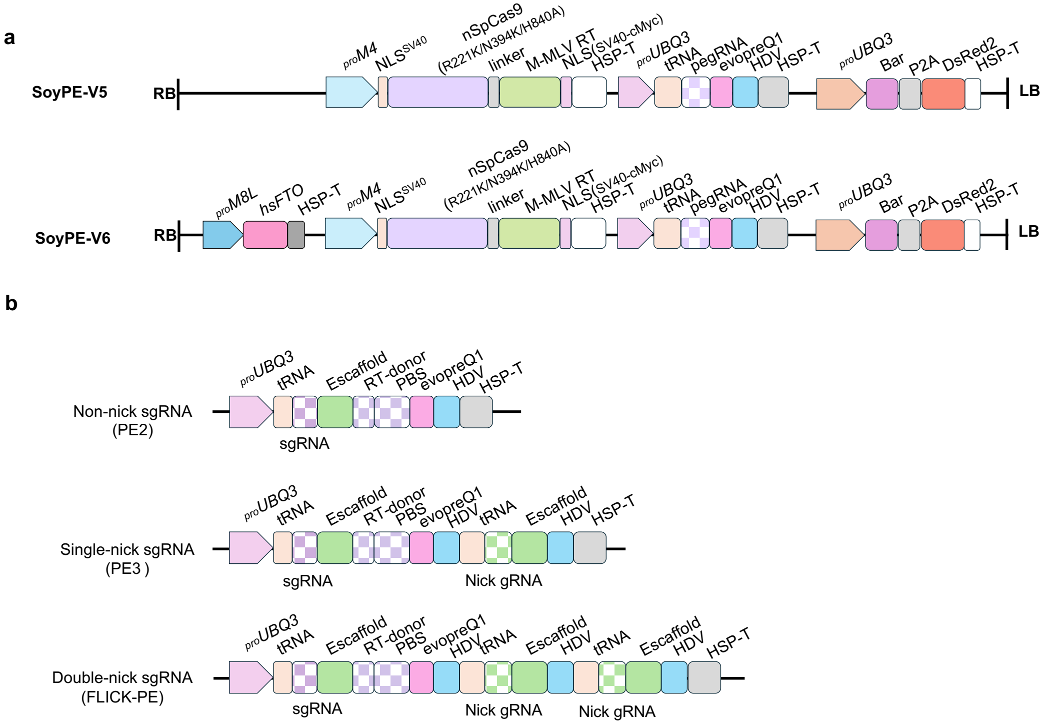


**Supplementary Fig. 1 | Vector structure for PE in soybean. a** PE vector structure for SoyPE-V5 from RB (right border) to LB (left border). **b** Schematic of pegRNA expression structures from top to bottom: PE2 with individually expressed pegRNA, PE3 incorporating a nick sgRNA, and FLICK-PE containing two nick sgRNAs**.**

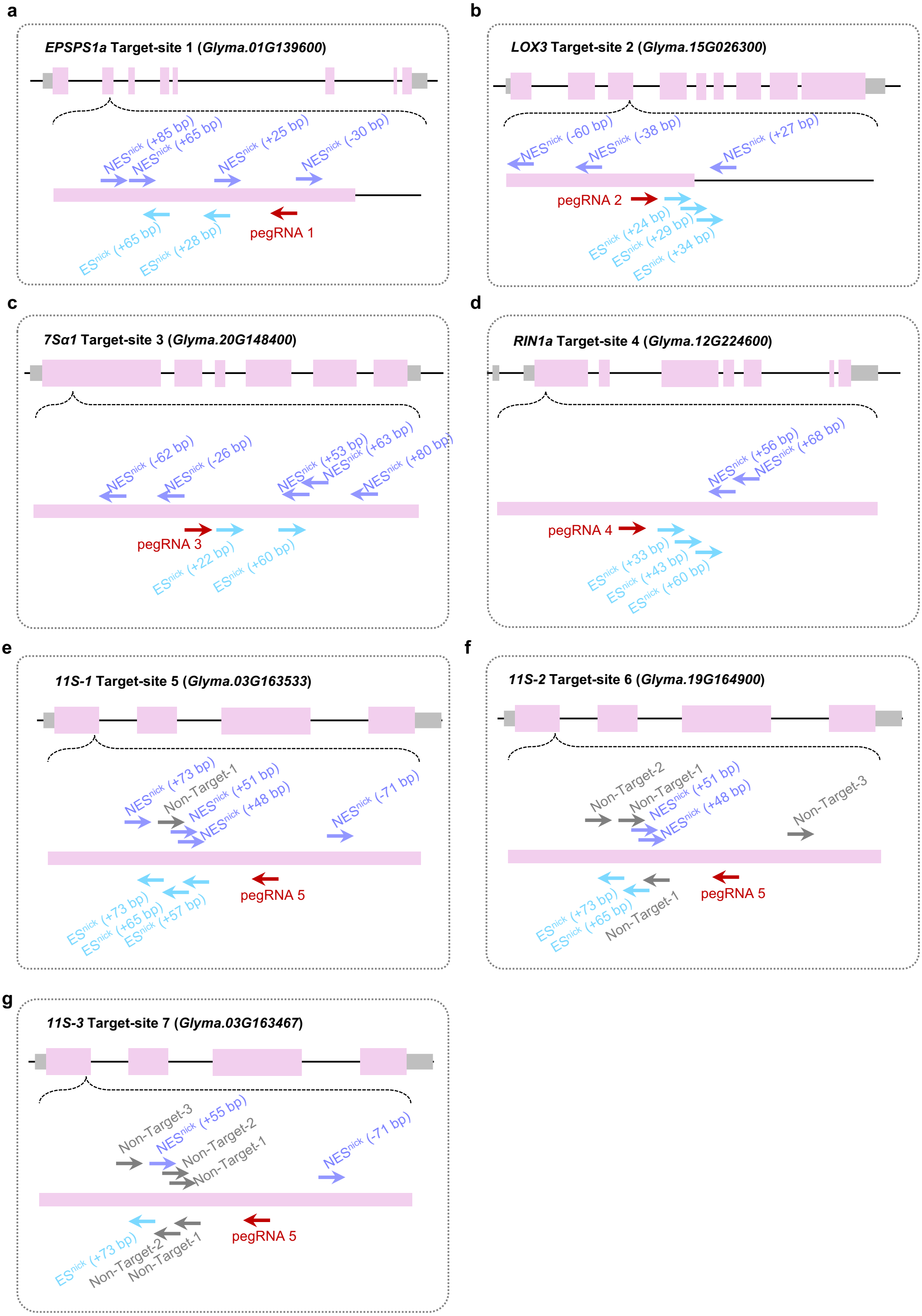
a
*EPSPS1a* Target-site 1 (*Glyma.01G139600*)
NES$^{nick}$ (+85 bp)
NES$^{nick}$ (+65 bp)
NES$^{nick}$ (+25 bp)
NES$^{nick}$ (-30 bp)
ES$^{nick}$ (+65 bp)
ES$^{nick}$ (+28 bp)
pegRNA 1
b
*LOX3* Target-site 2 (*Glyma.15G026300*)
NES$^{nick}$ (-60 bp)
NES$^{nick}$ (-38 bp)
NES$^{nick}$ (+27 bp)
pegRNA 2
ES$^{nick}$ (+24 bp)
ES$^{nick}$ (+29 bp)
ES$^{nick}$ (+34 bp)
c
*7Sα1* Target-site 3 (*Glyma.20G148400*)
NES$^{nick}$ (-62 bp)
NES$^{nick}$ (-26 bp)
NES$^{nick}$ (+53 bp)
NES$^{nick}$ (+63 bp)
NES$^{nick}$ (+80 bp)
pegRNA 3
ES$^{nick}$ (+22 bp)
ES$^{nick}$ (+60 bp)
d
*RIN1a* Target-site 4 (*Glyma.12G224600*)
NES$^{nick}$ (+56 bp)
NES$^{nick}$ (+68 bp)
pegRNA 4
ES$^{nick}$ (+33 bp)
ES$^{nick}$ (+43 bp)
ES$^{nick}$ (+60 bp)
e
*11S-1* Target-site 5 (*Glyma.03G163533*)
NES$^{nick}$ (+73 bp)
Non-Target-1
NES$^{nick}$ (+51 bp)
NES$^{nick}$ (+48 bp)
NES$^{nick}$ (-71 bp)
ES$^{nick}$ (+73 bp)
ES$^{nick}$ (+65 bp)
ES$^{nick}$ (+57 bp)
pegRNA 5
f
*11S-2* Target-site 6 (*Glyma.19G164900*)
Non-Target-2
Non-Target-1
NES$^{nick}$ (+51 bp)
NES$^{nick}$ (+48 bp)
Non-Target-3
ES$^{nick}$ (+73 bp)
ES$^{nick}$ (+65 bp)
Non-Target-1
pegRNA 5
g
*11S-3* Target-site 7 (*Glyma.03G163467*)
Non-Target-3
NES$^{nick}$ (+55 bp)
Non-Target-2
Non-Target-1
NES$^{nick}$ (-71 bp)
ES$^{nick}$ (+73 bp)
Non-Target-2
Non-Target-1
pegRNA 5

**Supplementary Fig. 2 | Design of target sites for testing PE strategies in soybean. a–g** Genomic structures of the indicated target genes, positions of pegRNA binding sites and nick sgRNA cleavage sites away from the pegRNA cleavage site. Each panel represents an individual target-site configuration evaluated for prime-editing efficiency at targets 1–7 in the soybean genome. Arrow directions indicate whether target sites reside on the sense or antisense DNA strand. Color-coded arrowheads specify functional attributes: red arrows denote pegRNA binding orientation, purple arrows mark nick sgRNA-mediated cleavage positions on the non-editing strand, blue arrows indicate nick sgRNA cleavage sites on the editing strand. Gray arrows highlight nick sgRNA designs containing sequence mismatches due to existing SNPs (single-nucleotide polymorphisms). Numeric labels quantify the distance (in base pairs) between nick sgRNA cleavage sites and the pegRNA-induced nick. Plus (+) and minus (–) symbols designate downstream or upstream positioning relative to the pegRNA cleavage sites, respectively.

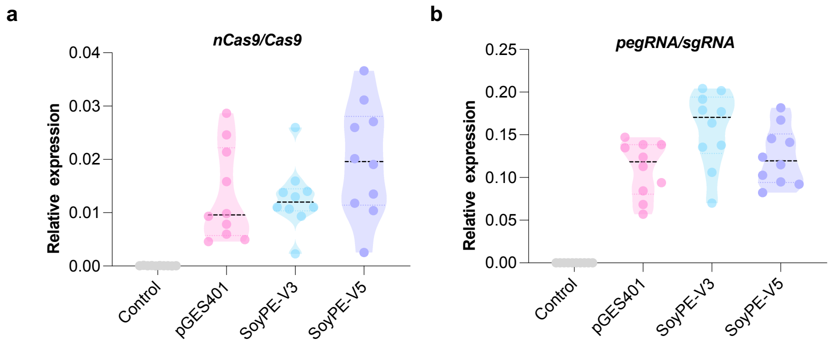


**Supplementary Fig. 3 | qRT–PCR of PE components expressed in transgenic soybean hairy roots. a** Relative expression levels of *nCas9* (H850A) and *Cas9*. Wild-type roots served as negative controls. The *Cas9* expression construct (pGES401) and two *nCas9* H804A variants (SoyPE-v3 and SoyPE-V5) were evaluated. **b** Relative expression levels of pegRNA and conventional sgRNA. Wild-type roots were used as baseline controls. The sgRNA-expressing vector (pGES401) and two pegRNA constructs (SoyPE-V3 and SoyPE-V5) were analyzed. Transcript levels of *nCas9*/*Cas9* and pegRNA/sgRNA were normalized to *Tefs* (10 hairy root samples were selected per test group, n = 10).

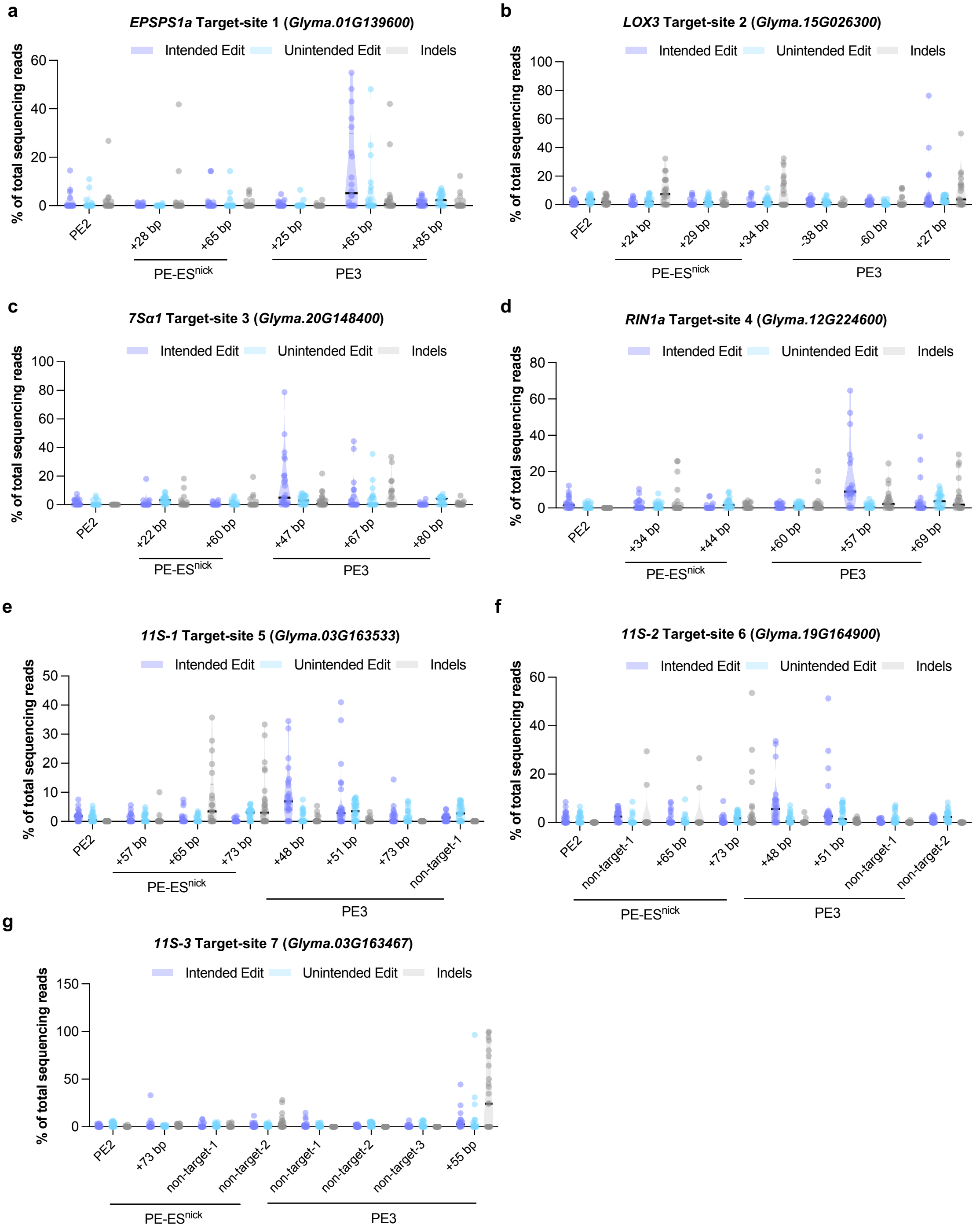


**Supplementary Fig. 4 | Editing efficiency mediated by PE3 and PE–ES$^{nick}$ in soybean hairy roots. a–g** Quantification of prime-editing outcomes across seven target loci (Target 1–7) mediated by PE3 and PE–ES$^{nick}$. Violin plots quantify editing precision: purple shows intended edits matching the intended modification, blue indicates unintended edits with unintended sequence changes, and gray depicts indels. Each panel corresponds to a distinct genomic locus and the central black lines in the violin plots represent the medians. The horizontal gray lines represent the lower and upper quartiles, and the shaded areas represent data-distribution density. The data presented are derived from three biological replicates, involving 20 (a–d) and 27 (e–g) hairy-root samples. All the data points are shown on the plots.

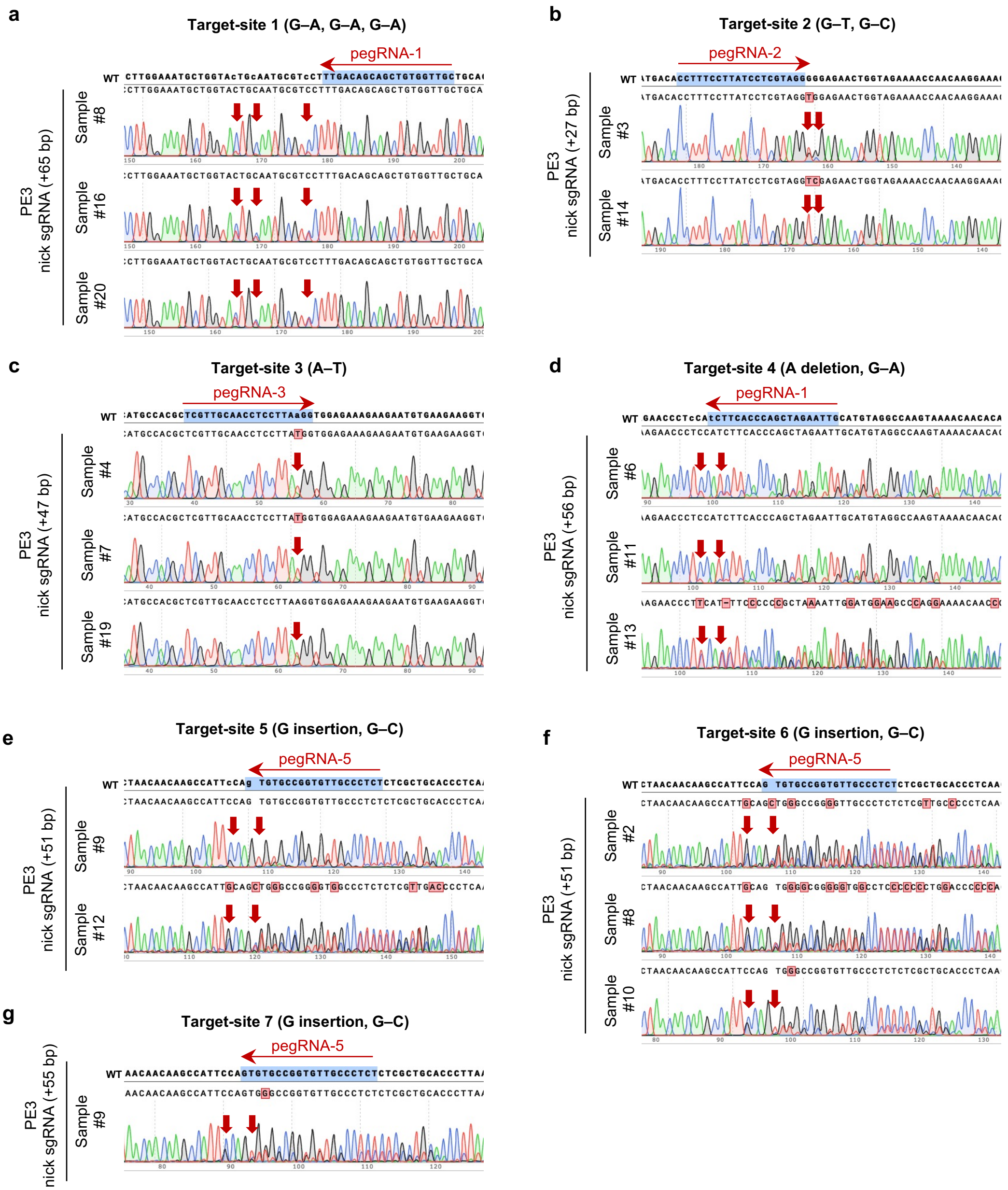


**Supplementary Fig. 5 | Representative chromatograms of PE3 mediated intended editing in soybean hairy roots. a–g** Sanger sequencing of editing outcomes at seven target loci (Target 1–7). Samples with ≥ 20% editing efficiency (quantified by Hi-TOM deep sequencing) were selected for validation. Red arrows highlight the intended modification sites. Double peaks observed in panels **(d–g)** are attributed to insertions or deletions (indels) introduced during intended editing. The bolded sequences at the top represent the wild-type sequences; the unbolded sequences above the peak plots denote the mutant sequences. Sequences with a blue background indicate the sgRNA sequences, and the direction of the red arrow signifies the orientation of the sgRNA.

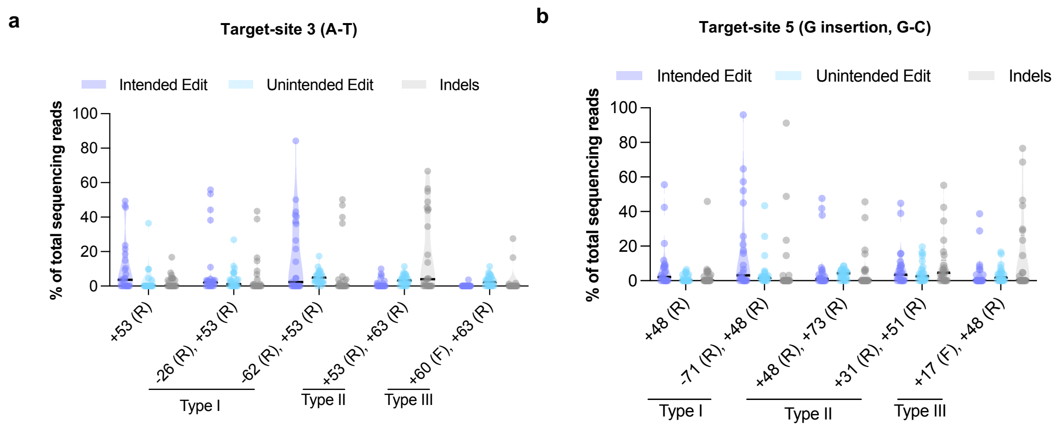


**Supplementary Fig. 6 | Editing efficiency mediated by dual-nicking strategies at Targets 3 and 5 in soybean hairy roots.** Quantification of PE editing outcomes across Target 1 **(a)** and Target 7 **(b)** mediated by PE–ES$^{nick}$ and PE3 strategies. Type I, II and III refer to FLICK-PE , asymmetric nicking and cross-strand nicking, respectively. Numeric labels quantify the distance (in base pairs) between nick sgRNA cleavage sites and the pegRNA-induced nick. Plus (+) and minus (–) symbols designate downstream or upstream positioning relative to the pegRNA cleavage sites, respectively. F and R represent the sgRNA targeting the editing strand or non-editing strand, respectively. Violin plots quantify editing precision: purple depicts intended edits matching the intended modification, blue for unintended edits with unintended sequence changes, and gray depicts indels. The central black lines in the violin plots represent the median, horizontal gray lines represent the lower and upper quartiles, and the shaded areas represent data distribution density **(a, b)**. Data presented are derived from three biological replicates, involving a total of 25 hairy root samples. All the data points are shown on the plots.

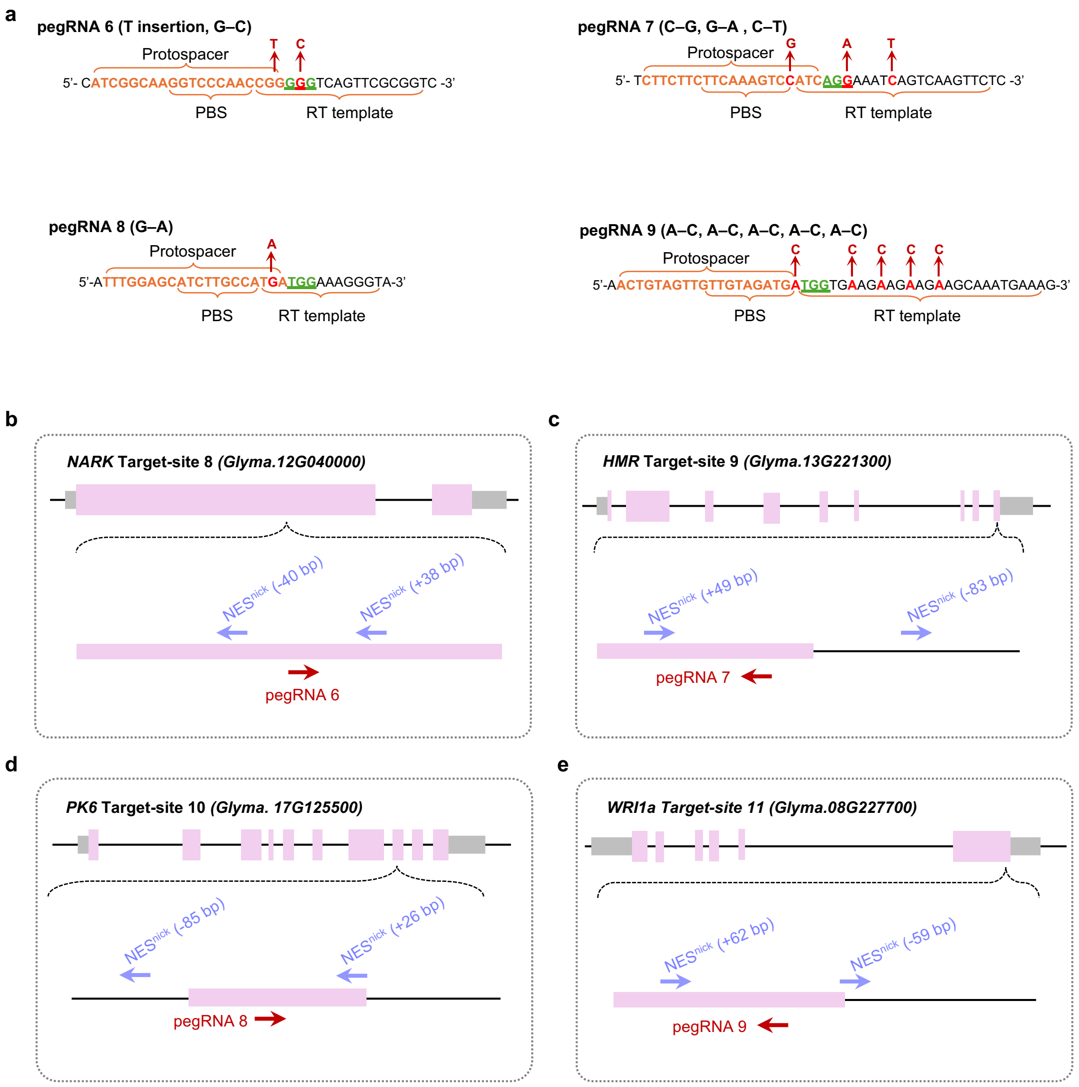


**Supplementary Fig. 7 | Design of new target sites for testing FLICK-PE in soybean. a** Design of pegRNA-6 to pegRNA-9. **b–e** Genomic structures and positioning of pegRNA binding sites and nick sgRNA cleavage sites away from the pegRNA cleavage site in the soybean genome. Each panel represents an individual target-site (Target 8 to Target 11) configurations evaluated for prime-editing efficiency. Arrow directions indicate whether target sites reside on the sense or antisense DNA strand. Color-coded arrowheads specify functional attributes: red arrows denote pegRNA binding orientation and purple arrows mark nick sgRNA-mediated cleavage positions on the non-editing strand. Numeric labels quantify the distance (in base pairs) between nick sgRNA cleavage sites and the pegRNA-induced nick. Plus (+) and minus (–) symbols designate downstream or upstream positioning relative to the pegRNA cleavage sites, respectively.

**a**

| pegRNA 1 | Sequence (5'-3') GCAACCACAGCTGCTGTCAA**AGG** |
|---|---|
| Off-T1 | GCAACTACAGCTGCTGTCAA**AGG** |
| Off-T2 | GCCACCACCTTTGCTGTCAA**AGG** |
| Off-T3 | GTAATCACAGCTGTTGTCAA**ACG** |
| Off-T4 | GCAACTATAGCTGCTGACAA**AGA** |
| Off-T5 | GCAACAACAGCTTTTGGCAA**AGG** |

**b**

| Nicking sgRNA 1 | Sequence (5'-3') GGGATTGTTTCCCACTAGTA**AGG** |
|---|---|
| Off-T1 | GGGATAGTTACCAACTAGTA**GGG** |
| Off-T2 | GGGATTGTTTCCCACTATTA**AAG** |
| Off-T3 | GGGATATTTACCAACTAGTA**GGG** |
| Off-T4 | GGGATTTTTTCCAACTAATG**TGG** |
| Off-T5 | GAGATTGTTACCCAATATTA**TGG** |

**c**

| Nicking sgRNA 2 | Sequence (5'-3') TTGCTGCAGGTGGAAATGCA**AGG** |
|---|---|
| Off-T1 | TTGATACAGTTGGAGATGC**GGG** |
| Off-T2 | AAGCTGCAGGAGGAAATGCT**GGG** |
| Off-T3 | TTGTTGAAATTGGAAATGCA**AGG** |
| Off-T4 | TTGATGCAGAGGAAAATGCA**AGG** |
| Off-T5 | CTACTGCAGTTCGAAATGCA**TGG** |

**d**

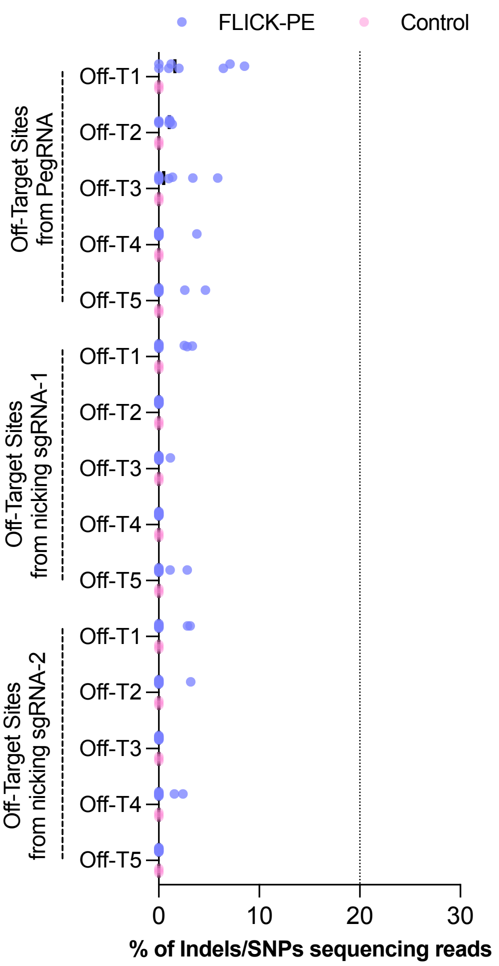


**Supplementary Fig. 8 | Evaluation of off-target effects from FLICK-PE in soybean hairy roots induced by pegRNA 1.** **a–c** Sequences of the top-five predicted off-target loci at pegRNA 1 (**a**), nicking sgRNA 1 (**b**) and nicking sgRNA 2 (**c**) predicted using CRISPR-GE. Red letters indicate that the bases at the corresponding positions in off-target sites exhibit SNP differences from those in the pegRNA, while black letters indicate that the bases at the corresponding positions in off-target sites match those at the same positions in the pegRNA. Bold and underlined sequences denote PAM sequences. **d** Percentage of Indels/SNPs generated at each off-target site relative to total reads was detected via Hi-TOM deep sequencing. The selection criteria for hairy-root samples with FLICK-PE were as follows: priority was given to samples with intended editing, followed by samples with unintended editing, and finally transgenic hairy roots without editing (n = 8); samples in the control group were non-transgenic hairy roots (n = 3).

**a**

| pegRNA 3 | **Sequence (5'-3')** TCGTTGCAACCTCCTTAAGG**TGG** |
|---|---|
| Off-T1 | TGGTTCCAACCTCCTTAAGA**GGG** |
| Off-T2 | TAGCTGCAATCTCCTTAAGC**TGG** |
| Off-T3 | CCGTTGCAACCTCCATAAGG**CAG** |
| Off-T4 | TGGCTGCACCATCCTTAAGG**GGG** |
| Off-T5 | TCTTTGCAACCTCCTAAAGT**TAG** |

**b**

| Nicking sgRNA 1 | **Sequence (5'-3')** CTCTCTCGCTATTGCAACTC**TGG** |
|---|---|
| Off-T1 | CACTCTAACTCTTGCAACTC**CGG** |
| Off-T2 | CTCTCTCTCTGCTGCAATTC**AGG** |
| Off-T3 | CTTTCTCGCTATTGCAACTT**CGG** |
| Off-T4 | CTCTCATGGTATTCCAACTC**CGG** |
| Off-T5 | CTTTGTCGCAATTGCAACTA**TGG** |

**c**

| Nicking sgRNA 2 | **Sequence (5'-3')** GGGTGTTGTGGTCGTGGTCG**TGG** |
|---|---|
| Off-T1 | GCGTGTAGTGGTCGTAGTCG**TGG** |
| Off-T2 | GGGTCTTTTAGTCGTGGTCG**TGG** |
| Off-T3 | GGGCGTAGTGATCATGGTCG**TGG** |
| Off-T4 | ATGTGTTGTGGTCGAAGTCG**TGG** |
| Off-T5 | AGGGGTGGTGGTCGTGGTCG**TGG** |

**d**

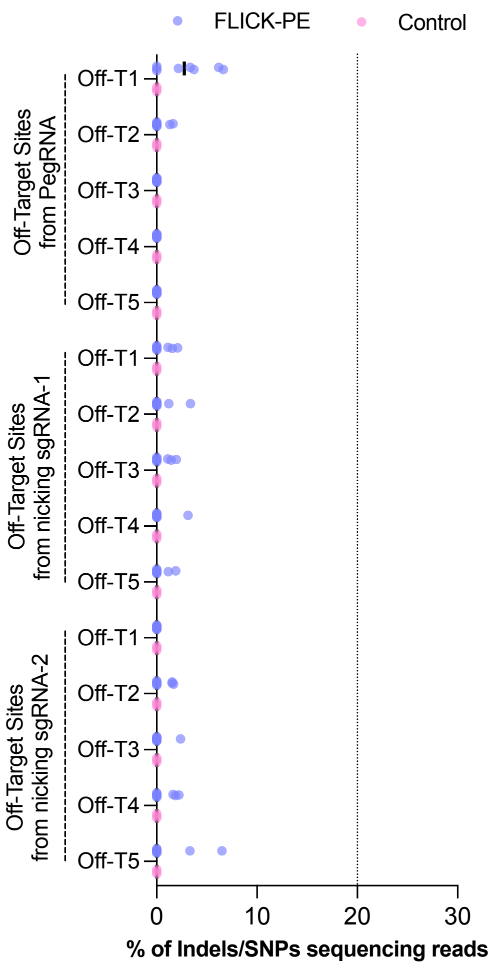


**Supplementary Fig. 9 | Evaluation of off-target effects from FLICK-PE in soybean hairy roots induced by pegRNA 3.** **a–c** Sequences of the top-five predicted off-target loci at pegRNA 3 (**a**), nicking sgRNA 1 (**b**) and nicking sgRNA 2 (**c**) predicted using CRISPR-GE. Red letters indicate that the bases at the corresponding positions in off-target sites exhibit SNP differences from those in the pegRNA, while black letters indicate that the bases at the corresponding positions in off-target sites match those at the same positions in the pegRNA. Bold and underlined sequences denote PAM sequences. **d** Percentage of Indels/SNPs generated at each off-target site relative to total reads detected via Hi-TOM deep sequencing. The selection criteria for hairy root samples under FLICK-PE were as follows: priority was given to samples with intended editing, followed by samples with unintended editing, and finally transgenic hairy roots without editing (n = 8); samples in the control group were non-transgenic hairy roots (n = 3).

a

| pegRNA 5 | Sequence (5'-3') AGAGGGCAACACCGGCACAC**TGG** |
|---|---|
| Off-T1 | AGAGATCAACACTGGCACAC**CGG** |
| Off-T2 | AGAGGGGAACAATGAAACAC**TGG** |
| Off-T3 | AGAGGTCAACACAGGCATAC**TGG** |
| Off-T4 | GGAGGGCAAAAGTGGCACTC**TGG** |
| Off-T5 | GGAAGGGAAAACCTGCACAC**TGG** |

b

| Nicking sgRNA 1 | Sequence (5'-3') AACCGTATAGAGTCAGAAGG**AGG** |
|---|---|
| Off-T1 | AACCGTATAGAGTCGGAAGG**TGG** |
| Off-T2 | AACTATATAGATTCAAAAGG**CGG** |
| Off-T3 | AATCCTATAAAGTCAGAGGG**AGG** |
| Off-T4 | AACCCTAGAGAGCAAGAAGG**GGG** |
| Off-T5 | AAAGGTTTAGGGTCAGAAGG**TGG** |

c

| Nicking sgRNA 2 | Sequence (5'-3') TTCCTACACCAACGGTCCCC**AGG** |
|---|---|
| Off-T1 | TTCCTACACCAACGCTCCCC**AGG** |
| Off-T2 | TTCCTACACTAACAGTCACA**GGG** |
| Off-T3 | TTCCTACAAGAACAGTCACC**GGG** |
| Off-T4 | TTCCTACACGGACAGTCACC**GGG** |
| Off-T5 | TTCCTACATCAACGTTCCTG**TGG** |

d

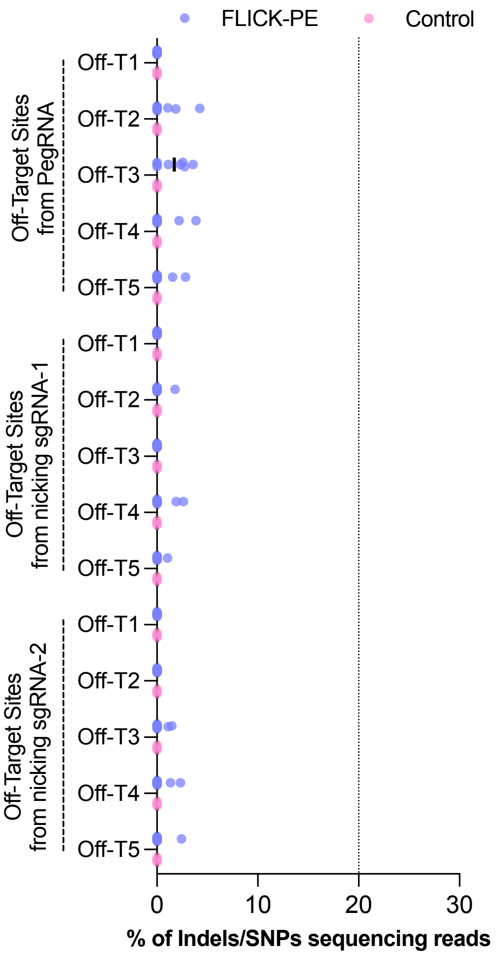


**Supplementary Fig. 10 | Evaluation of off-target effects from FLICK-PE in soybean hairy roots induced by pegRNA 5.** **a–c** Sequences of the top-five predicted off-target loci at pegRNA 5 (**a**), nicking sgRNA 1 (**b**) and nicking sgRNA 2 (**c**) predicted using CRISPR-GE. Red letters indicate that the bases at the corresponding positions in off-target sites exhibit SNP differences from those in the pegRNA, while black letters indicate that the bases at the corresponding positions in off-target sites match those at the same positions in the pegRNA. Bold and underlined sequences denote PAM sequences. **d** Percentage of Indels/SNPs generated at each off-target site relative to total reads detected via Hi-TOM deep sequencing. The selection criteria for hairy root samples under FLICK-PE were as follows: priority was given to samples with intended editing, followed by samples with unintended editing, and finally transgenic hairy roots without editing (n = 8); samples in the control group were non-transgenic hairy roots (n = 3).

**a**

| pegRNA 8 | Sequence (5'-3') ATCGGCAAGGTCCCAACCGG**GGG** |
|---|---|
| Off-T1 | ATCGGCAAGGTCCCTACCGG**TGG** |
| Off-T2 | ATAGGCAAGTTTCCAACTTG**GGG** |
| Off-T3 | ACAGGCAAGGTCCCCAACGA**GGG** |
| Off-T4 | ACCGGCATGGCCCAAACCAG**GGG** |
| Off-T5 | ATCGTCAGGGTCACAAGCGG**TGG** |

**b**

| Nicking sgRNA 1 | Sequence (5'-3') GAGGTTGGGGTTCCCTGCAA**AGG** |
|---|---|
| Off-T1 | GAGATTCGGGTTCCCTGCAA**AGG** |
| Off-T2 | GAGGTTCAGGTTCTCAGCAA**TGG** |
| Off-T3 | GAGGTTGAAATTCTCTGCAA**TGG** |
| Off-T4 | GAGGGTGTGTTTCCCTACAA**AGG** |
| Off-T5 | GAGATTGGGGTTACCAGCAG**AGG** |

**c**

| Nicking sgRNA 2 | Sequence (5'-3') GTTGTTGGATAGATCCAATG**TGG** |
|---|---|
| Off-T1 | TTTGTTGGAAAAATCCAATG**GGG** |
| Off-T2 | GTTATTAGACAGATCCAATG**GGG** |
| Off-T3 | GTTATTTGACAGATCCAATG**GGG** |
| Off-T4 | GTTGTTGAATACATCAAATG**GAG** |
| Off-T5 | TTTGTTGAATATTTCCAATG**AGG** |

**d**

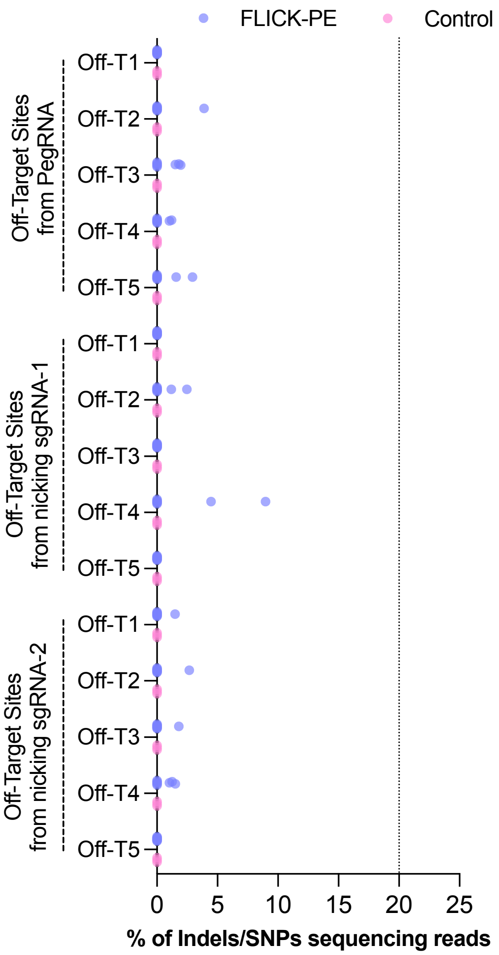


**Supplementary Fig.11 | Evaluation of off-target effects from FLICK-PE in soybean hairy roots induced by pegRNA 8.** **a–c** Sequences of the top-five predicted off-target loci at pegRNA 8 (**a**), nicking sgRNA 1 (**b**) and nicking sgRNA 2 (**c**) predicted using CRISPR-GE. Red letters indicate that the bases at the corresponding positions in off-target sites exhibit SNP differences from those in the pegRNA, while black letters indicate that the bases at the corresponding positions in off-target sites match those at the same positions in the pegRNA. Bold and underlined sequences denote PAM sequences. **d** Percentage of Indels/SNPs generated at each off-target site relative to total reads detected via Hi-TOM deep sequencing. The selection criteria for hairy root samples under FLICK-PE were as follows: priority was given to samples with intended editing, followed by samples with unintended editing, and finally transgenic hairy roots without editing (n = 8); samples in the control group were non-transgenic hairy roots (n = 3).

**a**

| pegRNA 9 | Sequence (5'-3') CTTCTTCTTCAAAGTCCATCAGG |
|---|---|
| Off-T1 | CTTCTTCTTCAAAGTCCATCAGA |
| Off-T2 | CTTCTTCTTCAATGTCTATCAAG |
| Off-T3 | CTTCTTCTTCAATGTCTATCAAG |
| Off-T4 | CTTCTTCTTCAACCTCCATCAGC |
| Off-T5 | CTTCTTCTTCAAAGTCCTTCATC |

**b**

| Nicking sgRNA 1 | Sequence (5'-3') GAAAGAAGGTCCTTTGTCACTGG |
|---|---|
| Off-T1 | GAAAAAAGGTACCTTGTCACAGG |
| Off-T2 | AAAAAAAGGTACCTTGTCACAGG |
| Off-T3 | AAAAAAAGGTCATTTGTAACCGG |
| Off-T4 | GAATTAAGGTCTTTTGCCATGG |
| Off-T5 | TGAAGAAGGTTCTTTGTCACAAG |

**c**

| Nicking sgRNA 2 | Sequence (5'-3') CATTGTGTAGACAGAATAGAAGG |
|---|---|
| Off-T1 | GATTGCATAGACAAAATAGACGG |
| Off-T2 | AATTGTATAAATAGAATAGAAGG |
| Off-T3 | CATTGTGTGGACAAAATAGATGG |
| Off-T4 | AATTGTATTGACAAAATAGATGG |
| Off-T5 | GAATGTGAAGACAAAATAGAAGG |

**d**

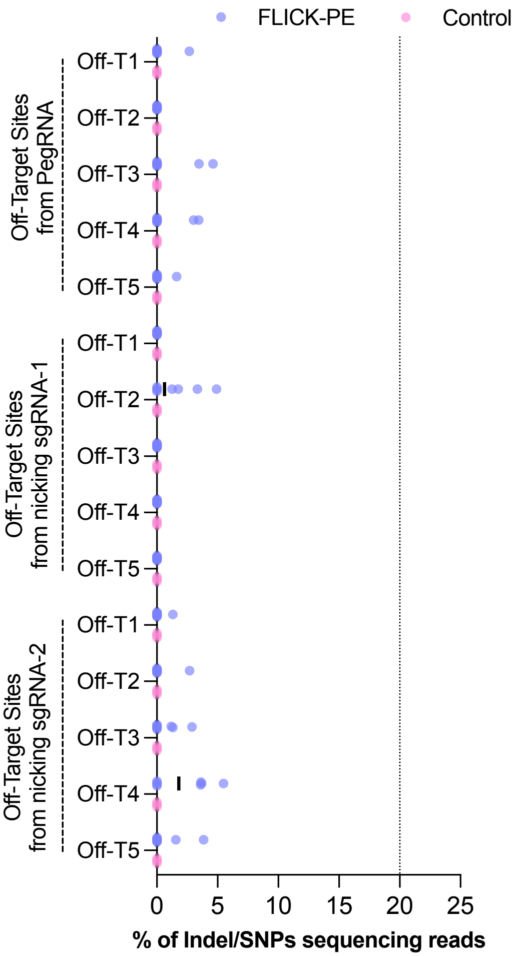


**Supplementary Fig. 12 | Evaluation of off-target effects from FLICK-PE in soybean hairy roots induced by pegRNA 9.** **a–c** Sequences of the top-five predicted off-target loci at pegRNA 9 (**a**), nicking sgRNA 1 (**b**) and nicking sgRNA 2 (**c**) predicted using CRISPR-GE. Red letters indicate that the bases at the corresponding positions in off-target sites exhibit SNP differences from those in the pegRNA, while black letters indicate that the bases at the corresponding positions in off-target sites match those at the same positions in the pegRNA. Bold and underlined sequences denote PAM sequences. **d** Percentage of Indels/SNPs generated at each off-target site relative to total reads detected via Hi-TOM deep sequencing. The selection criteria for hairy root samples under FLICK-PE were as follows: priority was given to samples with intended editing, followed by samples with unintended editing, and finally transgenic hairy roots without editing (n = 8); samples in the control group were non-transgenic hairy roots (n = 3).

a

| pegRNA 10 | Sequence (5’-3’) TTTGGAGCATCTTGCCATGA**TGG** |
|---|---|
| Off-T1 | TTTAGAGCATCTTGCAATGA**TGG** |
| Off-T2 | CTTGGAACATCTTGCCATGA**TGG** |
| Off-T3 | CTTGGAACATCTTGCCATGA**TGG** |
| Off-T4 | TTTAGAGCATCTGGCCATGA**TGG** |
| Off-T5 | TCTTGTGCATCTTGCCATGA**GGG** |

b

| Nicking sgRNA 1 | Sequence (5’-3’) TTCAACATGGGCTGTGGTAA**TGG** |
|---|---|
| Off-T1 | GTCAACATGGGTCGTGATAA**AGG** |
| Off-T2 | TTAAAAATGAGCTGTGGTAA**TAG** |
| Off-T3 | TTCAAAGTGGGTTGTGGTTA**AGG** |
| Off-T4 | TTCAGCATGGGATGTGATGA**AGG** |
| Off-T5 | TTTAACATCTGCTGTGGTAT**AGG** |

c

| Nicking sgRNA 2 | Sequence (5’-3’) ATGGAATAGAATCTAAGAGA**TGG** |
|---|---|
| Off-T1 | GTGGAATAGAACCTGAGAAA**TGG** |
| Off-T2 | GTGGAGTGGAAACTAAGAGA**AGG** |
| Off-T3 | ATGGAATAAAAACTAAGAAA**GGG** |
| Off-T4 | GTGGAATAGACACTAAGAGA**AGG** |
| Off-T5 | ATGCAAAAGAACTTAAGAGA**GGG** |

d

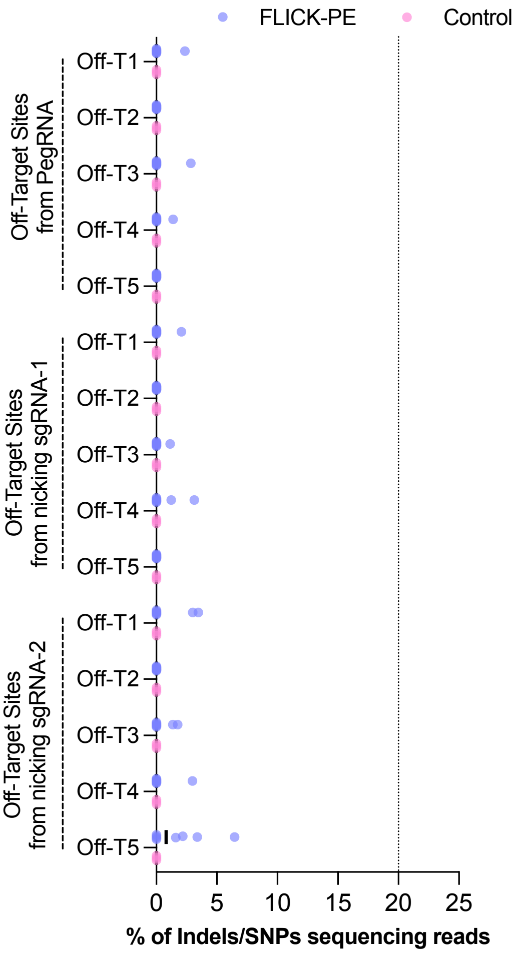


**Supplementary Fig. 13 | Evaluation of off-target effects from FLICK-PE in soybean hairy roots induced by pegRNA 10.** **a–c** Sequences of the top-five predicted off-target loci at pegRNA 10 (**a**), nicking sgRNA 1 (**b**) and nicking sgRNA 2 (**c**) predicted using CRISPR-GE. Red letters indicate that the bases at the corresponding positions in off-target sites exhibit SNP differences from those in the pegRNA, while black letters indicate that the bases at the corresponding positions in off-target sites match those at the same positions in the pegRNA. Bold and underlined sequences denote PAM sequences. **d** Percentage of Indels/SNPs generated at each off-target site relative to total reads detected via Hi-TOM deep sequencing. The selection criteria for hairy root samples under FLICK-PE were as follows: priority was given to samples with intended editing, followed by samples with unintended editing, and finally transgenic hairy roots without editing (n = 8); samples in the control group were non-transgenic hairy roots (n = 3).

**a**

| pegRNA 11 | Sequence (5'-3') ACTGTAGTTGTTGTAGATGA**TGG** |
|---|---|
| Off-T1 | TTTGTAGTTGTTGTAGATGA**TGG** |
| Off-T2 | TTTGTACTTGTTGTAGATGA**TGG** |
| Off-T3 | TCAGTAGATGTTGTAGATGG**TGG** |
| Off-T4 | ACTTTAGTTGCAGTAGATGA**TGG** |
| Off-T5 | CCTTTAGTTGTTGTAGATGT**TGG** |

**b**

| Nicking sgRNA 1 | Sequence (5'-3') AAGCATGGTGTATCATCATG**TGG** |
|---|---|
| Off-T1 | AAGCGTGGCTTATCAACATG**TGG** |
| Off-T2 | AAGAAAGTTGTATCATCATG**AGG** |
| Off-T3 | AAGAAAGCTGTATCATCATG**AGG** |
| Off-T4 | AAACAAGGTTTATTATCATG**AGG** |
| Off-T5 | AAGCATGTTACATCATCATT**TGG** |

**c**

| Nicking sgRNA 2 | Sequence (5'-3') TGATCTCTTCAGAAGGGTGT**TGG** |
|---|---|
| Off-T1 | TGATCACTTCAGAAAGGTGT**GGG** |
| Off-T2 | TGATCACCCCAGAAAGGTGT**GGG** |
| Off-T3 | TGATCACTTAAGAAAGGTGT**GGG** |
| Off-T4 | TGATCACTCCAAAAAGGTGT**GGG** |
| Off-T5 | TGATCACTCAAGAAAGGTGT**GGG** |

**d**

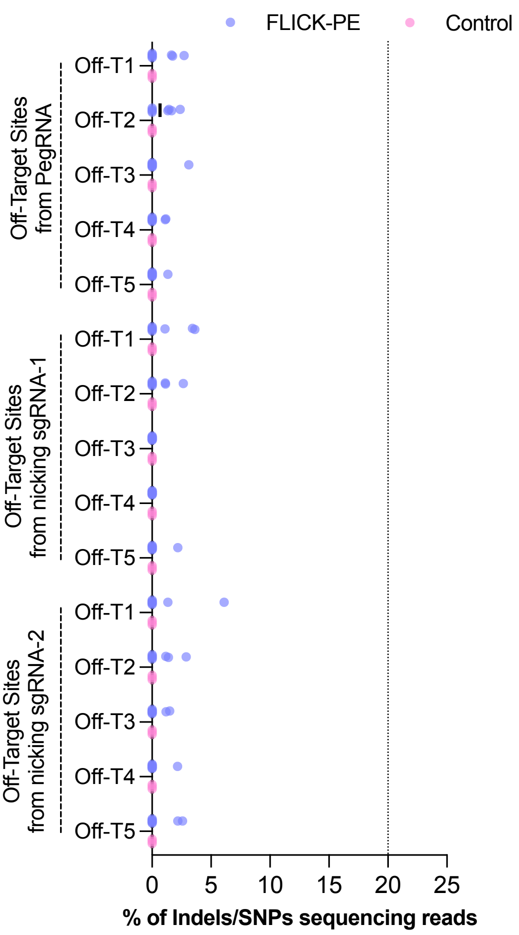


**Supplementary Fig. 14 | Evaluation of off-target effects from FLICK-PE in soybean hairy roots induced by pegRNA 11.** **a–c** Sequences of the top-five predicted off-target loci at pegRNA 11 (**a**), nicking sgRNA 1 (**b**) and nicking sgRNA 2 (**c**) predicted using CRISPR-GE. Red letters indicate that the bases at the corresponding positions in off-target sites exhibit SNP differences from those in the pegRNA, while black letters indicate that the bases at the corresponding positions in off-target sites match those at the same positions in the pegRNA. Bold and underlined sequences denote PAM sequences. **d** Percentage of Indels/SNPs generated at each off-target site relative to total reads detected via Hi-TOM deep sequencing. The selection criteria for hairy root samples under FLICK-PE were as follows: priority was given to samples with intended editing, followed by samples with unintended editing, and finally transgenic hairy roots without editing (n = 8); samples in the control group were non-transgenic hairy roots (n = 3).

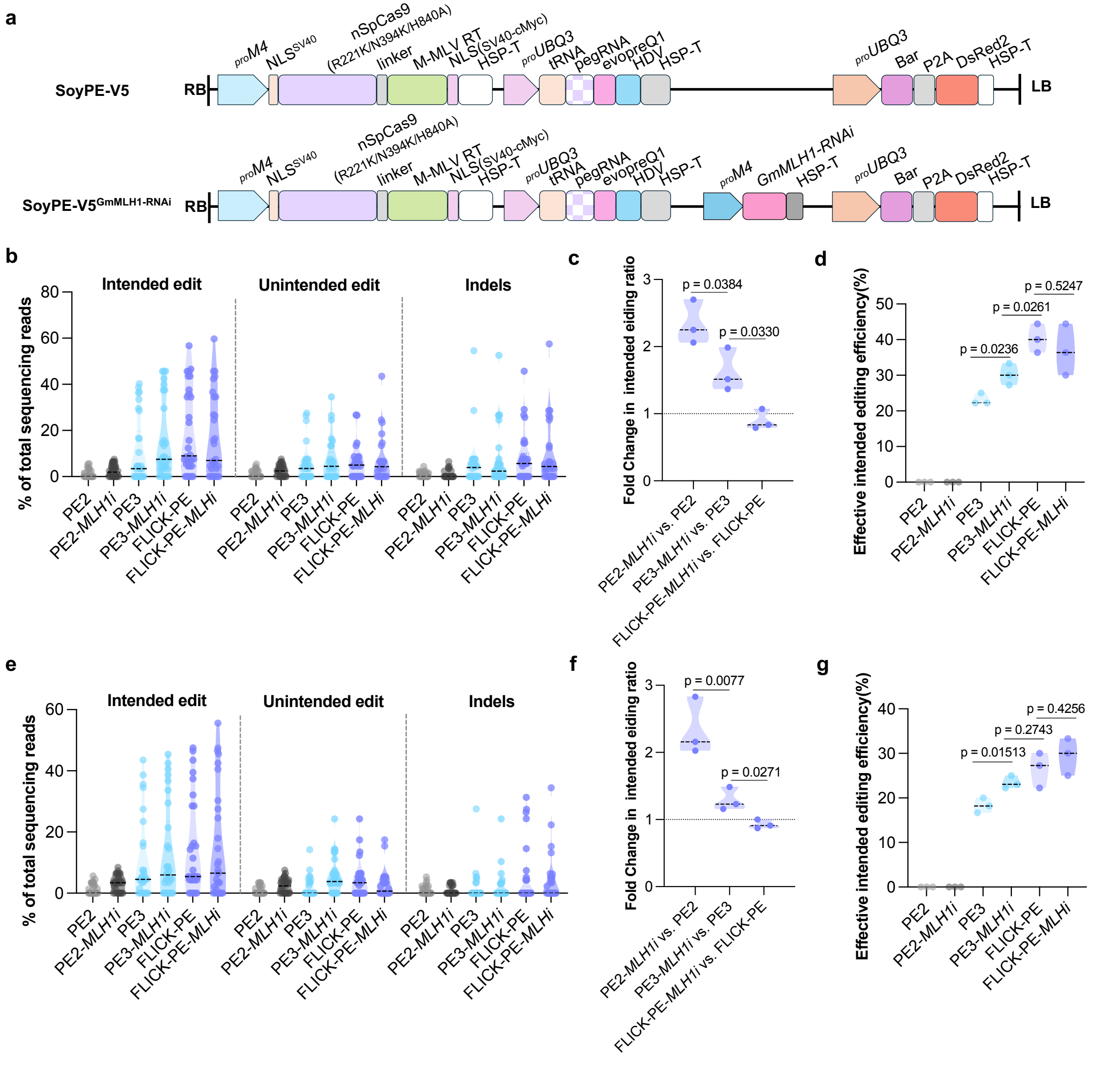


**Supplementary Fig. 15 | FLICK-PE relieves the inhibitory effect of the MMR pathway on PE efficiency. a** Schematic of PE vectors co-expressing the *GmMLH1* RNAi construct for soybean: The upper panel shows the control vector SoyPE-V5 and the lower panel shows the PE vector fused with an RNAi cassette to down-regulated *GmMLH1* expression. The RNAi expression cassette uses the *pM4* promoter and *HSP-T* terminator. **b**, **e** Editing efficiency at Target-site 1 (**b**) and Target-site 3 (**e**) in soybean hairy roots between *GmMLH1* expression-inhibited groups and the control vector: PE2-*MLH1i*, PE3-*MLH1i*, and FLICK-PE-*MLH1i* are all strategies fused with the *GmMLH1 RNAi* cassette, with PE2, PE3 and FLICK-PE serving as control vectors, respectively. **c**, **f** Comparison of fold changes in the percentage of intended editing (relative to total reads) per hairy root at Target-site 1 (**c**) and Target-site 3 (**f**) between each editing strategy (co-expressing the *GmMLH1* RNAi cassette) and the control vector. **d**, **g** Effective intended editing efficiency of all PE strategies (defined as the proportion of hairy roots with intended editing ratio > 20% by Hi-TOM deep sequencing among all tested roots) at Target-site 1 (**d**) and Target-site 3 (**g**). All the data points are shown on the plots. The central black lines in the violin plots represent the median in **(b–g)**. The horizontal gray lines represent the lower and upper quartiles, and the shaded areas represent data-distribution density in **(b–g)**. Data in in **(c, d, f, g, )** were analyzed by Two-tailed Mann–Whitney testing ($P < 0.05$). The data are derived from three biological replicates, involving 30 hairy-root samples. All the data points are shown on the plots.

a

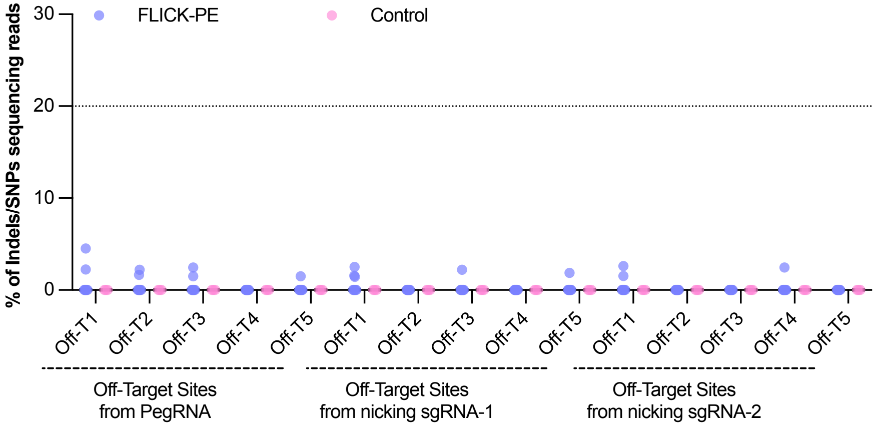


b

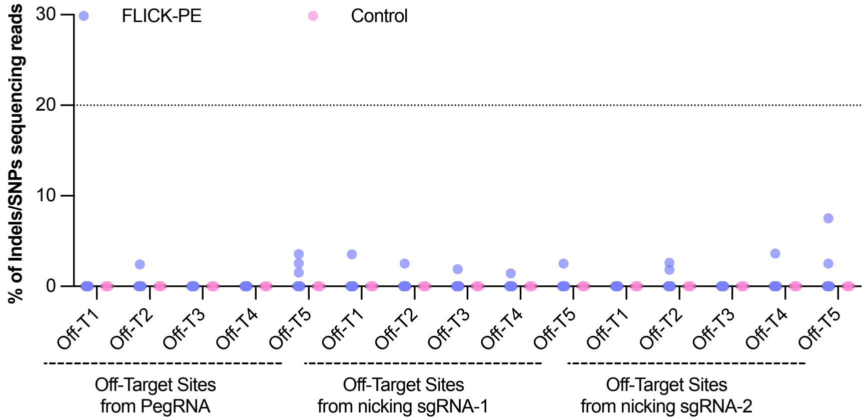


c

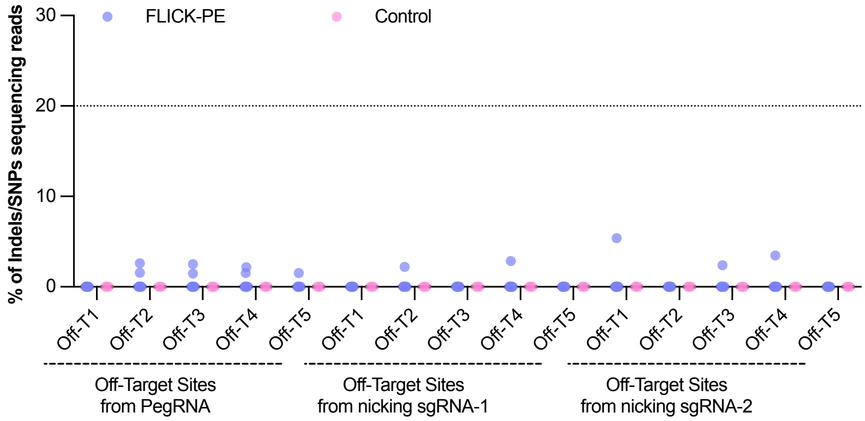


**Supplementary Fig. 16 | Evaluation of off-target effects from FLICK-PE in soybean stable transformation.** For pegRNA 1 (**a**), pegRNA 3 (**b**), and pegRNA 5 (**c**), the percentage of indels/SNPs at each off-target site relative to total reads was determined via Hi-TOM deep sequencing. For transgenic plants under FLICK-PE , selection criteria were: prioritizing plants with intended editing, followed by those with unintended editing, and finally transgenic plants without editing (n = 8); the control group consisted of wild-type soybean plants (n = 3).

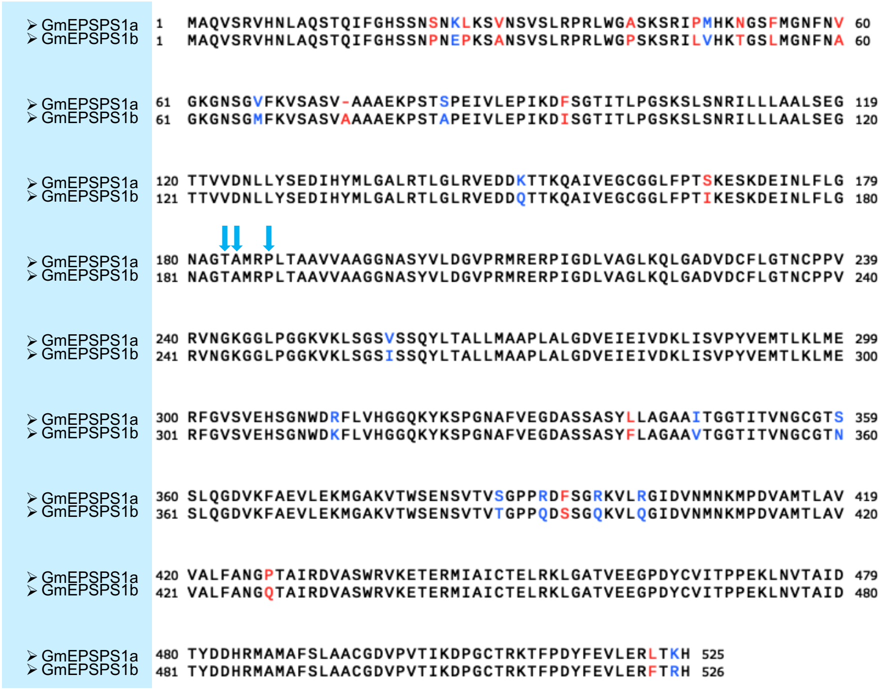

**Supplementary Fig. 17 | Amino-acid sequence alignment of soybean EPSPS homologs EPSPS1a and EPSPS1b.** Amino-acid sequence alignment of soybean EPSPS homologs EPSPS1a (*Glyma.01G139600*) and EPSPS1b (*Glyma.03G027400*). Blue arrows indicate intended editing sites, black letters represent identical amino acids, blue letters indicate similar amino acids, and red letters denote dissimilar amino acids.

**a**

**Target-site 1 *(Nbe02g16450)***

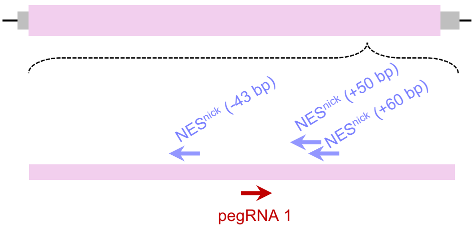


| Vector Name | pegRNA Name | Nick-sgRNA-A | Nick sgRNA-B |
|---|---|---|---|
| **non-nick** | pegRNA 1 | / | / |
| **PE3-1 (PE-NES$^{nick}$-1)** | pegRNA 1 | NES$^{nick}$(+50 bp) | / |
| **FLICK-PE-1** | pegRNA 1 | NES$^{nick}$(+50 bp) | NES$^{nick}$(-43 bp) |
| **PE3-2 (PE-NES$^{nick}$-2)** | pegRNA 1 | NES$^{nick}$(+60 bp) | / |
| **FLICK-PE-2** | pegRNA 1 | NES$^{nick}$(+60 bp) | NES$^{nick}$(-43 bp) |

**b**

**Target-site 2 *(Nbe02g24410)***

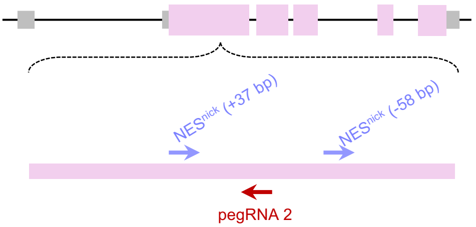


| Vector Name | pegRNA Name | Nick-sgRNA-A | Nick sgRNA-B |
|---|---|---|---|
| **non-nick** | pegRNA 2 | / | / |
| **PE3 (PE-NES$^{nick}$)** | pegRNA 2 | NES$^{nick}$(+37 bp) | / |
| **FLICK-PE** | pegRNA 2 | NES$^{nick}$(+37 bp) | NES$^{nick}$(-58 bp) |

**c**

**Target-site 3 *(Nbe02g16450)***

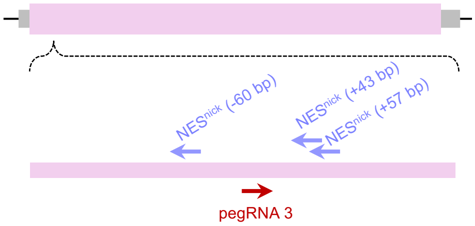


| Vector Name | pegRNA Name | Nick-sgRNA-A | Nick sgRNA-B |
|---|---|---|---|
| **non-nick** | pegRNA 3 | / | / |
| **PE3-1 (PE-NES$^{nick}$-1)** | pegRNA 3 | NES$^{nick}$(+57 bp) | / |
| **FLICK-PE-1** | pegRNA 3 | NES$^{nick}$(+57 bp) | NES$^{nick}$(-60 bp) |
| **PE3-2 (PE-NES$^{nick}$-2)** | pegRNA 3 | NES$^{nick}$(+43 bp) | / |
| **FLICK-PE-2** | pegRNA 3 | NES$^{nick}$(+43 bp) | NES$^{nick}$(-60 bp) |

**d**

**Target-site 4 *(Nbe02g16450)***

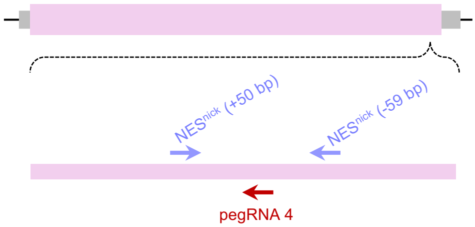


| Vector Name | pegRNA Name | Nick-sgRNA-A | Nick sgRNA-B |
|---|---|---|---|
| **non-nick** | pegRNA 4 | / | / |
| **PE3 (PE-NES$^{nick}$)** | pegRNA 4 | NES$^{nick}$(+50 bp) | / |
| **FLICK-PE** | pegRNA 4 | NES$^{nick}$(+50 bp) | NES$^{nick}$(-59 bp) |

**Supplementary Fig. 18 | Design of target sites for testing PE strategies in tobacco. a–d** Genomic structures of target genes, positions of pegRNA binding sites and nick sgRNA cleavage sites away from the pegRNA cleavage site. Each panel represents an individual target-site configuration (Targets 1–4) evaluated for prime-editing efficiency in the tobacco genome. Arrow directions indicate whether target sites reside on the sense or antisense DNA strand. Color-coded arrowheads specify functional attributes: red arrows denote pegRNA binding orientation and purple arrows mark nick sgRNA-mediated cleavage positions on the non-editing strand. Numeric labels quantify the distance (in base pairs) between nick sgRNA cleavage sites and the pegRNA-induced nick. Plus (+) and minus (–) symbols designate downstream or upstream positioning relative to the pegRNA cleavage sites, respectively.
