# Supplementary material for: A flanking-nicks prime editor (FLICK-PE) system to boost prime editing in dicots
Source: Nat Commun. 2025 Dec 4;17:337. doi: 10.1038/s41467-025-67046-3 (PMC12789627; doi:10.1038/s41467-025-67046-3)
Supplement: Supplementary file 2 — Description of Additional Supplementary Files [file 41467_2025_67046_MOESM2_ESM.pdf]

Description of Additional Supplementary Files

File Name: Supplementary Data 1
Description: pegRNA sequences for prime editing in soybean.

File Name: Supplementary Data 2
Description: Nick sgRNAs for prime editing in soybean.

File Name: Supplementary Data 3
Description: pegRNA sequences for prime editing in tobacco.

File Name: Supplementary Data 4
Description: Nick sgRNAs for prime editing in tobacco.

File Name: Supplementary Data 5
Description: Primers for Hi-TOM deep sequencing and Sanger sequencing.

File Name: Supplementary Data 6
Description: Primers for Q-RT-PCR.

File Name: Supplementary Data 7
Description: Sequence of the key elements of vectors.
