# Supplementary material for: A flanking-nicks prime editor (FLICK-PE) system to boost prime editing in dicots
Source: Nat Commun. 2025 Dec 4;17:337. doi: 10.1038/s41467-025-67046-3 (PMC12789627; doi:10.1038/s41467-025-67046-3)
Supplement: Supplementary file 5 — Reporting Summary [file 41467_2025_67046_MOESM5_ESM.pdf]

nature portfolio

Corresponding author(s): Yuefeng Guan

Last updated by author(s): Oct 30, 2025

Reporting Summary

Nature Portfolio wishes to improve the reproducibility of the work that we publish. This form provides structure for consistency and transparency in reporting. For further information on Nature Portfolio policies, see our Editorial Policies and the Editorial Policy Checklist.

Statistics

For all statistical analyses, confirm that the following items are present in the figure legend, table legend, main text, or Methods section.

n/a	Confirmed	
☐	☒	The exact sample size (*n*) for each experimental group/condition, given as a discrete number and unit of measurement
☐	☒	A statement on whether measurements were taken from distinct samples or whether the same sample was measured repeatedly
☐	☒	The statistical test(s) used AND whether they are one- or two-sided<br>*Only common tests should be described solely by name; describe more complex techniques in the Methods section.*
☒	☐	A description of all covariates tested
☒	☐	A description of any assumptions or corrections, such as tests of normality and adjustment for multiple comparisons
☐	☒	A full description of the statistical parameters including central tendency (e.g. means) or other basic estimates (e.g. regression coefficient) AND variation (e.g. standard deviation) or associated estimates of uncertainty (e.g. confidence intervals)
☐	☒	For null hypothesis testing, the test statistic (e.g. *F*, *t*, *r*) with confidence intervals, effect sizes, degrees of freedom and *P* value noted<br>*Give P values as exact values whenever suitable.*
☒	☐	For Bayesian analysis, information on the choice of priors and Markov chain Monte Carlo settings
☒	☐	For hierarchical and complex designs, identification of the appropriate level for tests and full reporting of outcomes
☒	☐	Estimates of effect sizes (e.g. Cohen's *d*, Pearson's *r*), indicating how they were calculated

Our web collection on statistics for biologists contains articles on many of the points above.

Software and code

Policy information about availability of computer code

Data collection	Illumina HiSeq X Ten platform was used to collect the Hi-TOM deep sequencing data
Data analysis	Data analysis was performed using the Hi-TOM online tool (http://www.hi-tom.net/hi-tom/index-CH.php). For all graphical representations and statistical analyses, Microsoft Excel 365 (Microsoft Corp., USA) was used for initial data processing and GraphPad Prism v10.0 (GraphPad Software, USA) was used for statistical analysis and visualization. The length of the RT template and PBS in each pegRNA was designed using PlantPegDesigner v1.0(Lin et al., 2021) (http://www.plantgenomeediting.net). Potential off-target sites were predicted using CRISPR-GE(Xie et al., 2017) (http://skl.scau.edu.cn/).

For manuscripts utilizing custom algorithms or software that are central to the research but not yet described in published literature, software must be made available to editors and reviewers. We strongly encourage code deposition in a community repository (e.g. GitHub). See the Nature Portfolio guidelines for submitting code & software for further information.

Data

Policy information about availability of data

All manuscripts must include a data availability statement. This statement should provide the following information, where applicable:

- Accession codes, unique identifiers, or web links for publicly available datasets
- A description of any restrictions on data availability
- For clinical datasets or third party data, please ensure that the statement adheres to our policy

All sequencing data have been deposited to China National Genomics Data Center under accession PRJCA017278.

Research involving human participants, their data, or biological material

Policy information about studies with human participants or human data. See also policy information about sex, gender (identity/presentation), and sexual orientation and race, ethnicity and racism.

Reporting on sex and gender	N/A
Reporting on race, ethnicity, or other socially relevant groupings	N/A
Population characteristics	N/A
Recruitment	N/A
Ethics oversight	N/A

Note that full information on the approval of the study protocol must also be provided in the manuscript.

Field-specific reporting

Please select the one below that is the best fit for your research. If you are not sure, read the appropriate sections before making your selection.

☒ Life sciences ☐ Behavioural & social sciences ☐ Ecological, evolutionary & environmental sciences

For a reference copy of the document with all sections, see nature.com/documents/nr-reporting-summary-flat.pdf

Life sciences study design

All studies must disclose on these points even when the disclosure is negative.

Sample size	For soybean hairy roots, one positive hairy root was treated as an independent sample with 10–15 samples per biological replicate, while soybean stable-transgenic plants and tobacco stable-transgenic plants used at least three leaves per plant as one sample. Transient tobacco transformation used one infiltrated leaf segment as one sample with 2–3 leaves per replicate.
Data exclusions	No data exclusion
Replication	All attempts for replication were successful. For the experiments in soybean hairy root, tobacco infiltration as sample was performed in three biological replicates.
Randomization	The seeds of soybean were prepared and randomly separated to each hairy root transformation. In transient tobacco transformation, to ensure the randomness of the experimental design, each vector was used to infiltrate different leaves of 3 to 5 plants selected at random. Meanwhile, different leaves of the same plant were co-infiltrated with multiple distinct vectors simultaneously.
Blinding	Not applicable. Transformation and sampling were performed consistently using standardized protocols (including transformation, sampling, plant selection, gDNA isolation, and DNA sequencing), which are designed to eliminate bias

Reporting for specific materials, systems and methods

We require information from authors about some types of materials, experimental systems and methods used in many studies. Here, indicate whether each material, system or method listed is relevant to your study. If you are not sure if a list item applies to your research, read the appropriate section before selecting a response.

Materials & experimental systems

n/a	Involved in the study
☒	☐ Antibodies
☒	☐ Eukaryotic cell lines
☒	☐ Palaeontology and archaeology
☒	☐ Animals and other organisms
☒	☐ Clinical data
☒	☐ Dual use research of concern
☐	☒ Plants

Methods

n/a	Involved in the study
☒	☐ ChIP-seq
☒	☐ Flow cytometry
☒	☐ MRI-based neuroimaging

Dual use research of concern

Policy information about dual use research of concern

Hazards

Could the accidental, deliberate or reckless misuse of agents or technologies generated in the work, or the application of information presented in the manuscript, pose a threat to:

No	Yes
☒	☐ Public health
☒	☐ National security
☒	☐ Crops and/or livestock
☒	☐ Ecosystems
☒	☐ Any other significant area

Experiments of concern

Does the work involve any of these experiments of concern:

No	Yes
☒	☐ Demonstrate how to render a vaccine ineffective
☒	☐ Confer resistance to therapeutically useful antibiotics or antiviral agents
☒	☐ Enhance the virulence of a pathogen or render a nonpathogen virulent
☒	☐ Increase transmissibility of a pathogen
☒	☐ Alter the host range of a pathogen
☒	☐ Enable evasion of diagnostic/detection modalities
☒	☐ Enable the weaponization of a biological agent or toxin
☒	☐ Any other potentially harmful combination of experiments and agents

Plants

Seed stocks	Soybean (Glycine max) cultivars William 82 (for hairy-root transformation) and Hua-Chun 6 (HC-6, for whole-plant stable transformation) were used, alongside tobacco (Nicotiana benthamiana).
Novel plant genotypes	The prime editing system was employed to introduce targeted genomic modifications into soybean and tobacco. Agrobacterium-mediated transformation was utilized as the delivery method for the prime editing components. The endogenous target sequences and targeting guide RNA sequences are detailed in Supplementary Data 1-5.
Authentication	No seed stock authentication was performed. The EPSPS1a (TAP-IVS) genotype was evaluated for glyphosate resistance in lab-scale assays. T-DNA insertion was assessed using dsRED2 on the vector backbone, and agronomic traits were evaluated in field trials. All generated seeds and materials were handled in accordance with standard laboratory protocols.
